# Females with Diabetes Mellitus Increased the Incidence of Premenstrual Syndrome

**DOI:** 10.3390/life12060777

**Published:** 2022-05-24

**Authors:** Yao-Ming Huang, Wu-Chien Chien, Chun-Gu Cheng, Yin-Han Chang, Chi-Hsiang Chung, Chun-An Cheng

**Affiliations:** 1 Department of Emergency Medicine, Taoyuan Armed Forces General Hospital, National Defense Medical Center, Taoyuan 32549, Taiwan; algernon8149@gmail.com (Y.-M.H.); doc50015@yahoo.com.tw (C.-G.C.); 2 Department of Medical Research, Tri-Service General Hospital, National Defense Medical Center, Taipei 11490, Taiwan; chienwu@ndmctsgh.edu.tw (W.-C.C.); g694810042@gmail.com (C.-H.C.); 3 School of Public Health, National Defense Medical Center, Taipei 11490, Taiwan; 4 Graduate Institute of Life Sciences, National Defense Medical Center, Taipei 11490, Taiwan; 5 Department of Emergency Medicine, Tri-Service General Hospital, National Defense Medical Center, Taipei 11490, Taiwan; 6 Emergency Department, Department of Emergency and Critical Medicine, Wan Fang Hospital, Taipei Medical University, Taipei 11696, Taiwan; 7 Department of Emergency, School of Medicine, College of Medicine, Taipei Medical University, Taipei 11031, Taiwan; 8 Department of Psychology, National Taiwan University, Taipei 10621, Taiwan; caalice2003@yahoo.com.tw; 9 Department of Neurology, Tri-Service General Hospital, National Defense Medical Center, Taipei 11490, Taiwan

**Keywords:** diabetes mellitus, premenstrual syndrome, autonomic dysfunction

## Abstract

Background: Premenstrual syndrome (PMS) is a multifactorial disorder caused by hormone and autonomic imbalance. In our study, hyperglycemia-induced insulin secretion increased progesterone secretion and progressive autonomic imbalance. The young patients with diabetes mellitus (DM) revealed hypo-parasympathetic function and hypersympathetic function compared with nondiabetic controls. Young female patients with DM with higher blood sugar and autonomic malfunction may be associated with PMS. However, there is a lack of evidence about DM in females related to PMS. We evaluated female patients with DM who subsequently followed PMS in a retrospective cohort study. Methods: We retrieved data from the National Health Insurance Research Database in Taiwan. Female patients with DM between 20 and 50 years old were assessed by the International Classification of Disease, 9 Revision, Clinical Modification (ICD-9-CM) disease code of 250. Patients who were DM-free females were fourfold matched to the control group by age and disease index date. The ICD-9-CM disease code of 625.4 identified the incidence of PMS followed by the index date as events. The possible risk factors associated with PMS were detected with a Cox proportional regression. Results: DM was a significant risk factor for PMS incidence with an adjusted hazard ratio of 1.683 (95% confidence interval: 1.104–2.124, *p* < 0.001) in females after adjusting for age, other comorbidities, season, urbanization status of patients and the hospital status of visiting. Conclusions: This study noted an association between DM and PMS in female patients. Healthcare providers and female patients with DM must be aware of possible complications of PMS, aggressive glycemic control, decreased hyperglycemia and autonomic dysfunction to prevent this bothersome disorder.

## 1. Introduction

Premenstrual syndrome (PMS) is an annoying disorder in normal cyclic females of possible childbearing status which affects their quality of life. Somatic and emotional problems occur during the luteal phase and until the next menstrual cycle begins, patients suffering from severe symptoms miss work or school. The etiologies of PMS are not fully understood [1]. Hormonal fluctuations cause the symptoms of PMS to disappear during pregnancy and menopause. The insufficient serotonin-enhanced sensitivity of progesterone affects PMS [2], but the blood sugar and autonomic system also seem to have some contributions. Smoking and high calorie/sugar food consumption among female university students resulted in a higher prevalence of PMS [3]. The evaluation of food intake and PMS symptoms in two menstrual cycles revealed sucrose preference and higher PMS scores [4]. Insulin stimulated by hyperglycemia could modulate the gonadotropin-releasing hormone (GnRH) receptors and increase LH secretion, and insulin and LH enhanced corpus luteum steroidogenesis with progesterone secretion [5,6].

Past studies found hypo-parasympathetic and sympathetic overactivity, and postural induced low baroreflex sensitivity, as well as higher low-frequency blood pressure variability in the late luteal phase [7,8]. A previous study found that palmar hyperhidrosis patients had an increased PMS incidence which may be related to autonomic dysfunction [9]. A change in eating habits increases the prevalence of DM at a young age. It is worth noting the conditions that can develop due to such a change. A past study showed that young patients with DM revealed hypo-parasympathetic function with total heart rate variability (HRV), decreased high-frequency HRV and increased hypersympathetic function with low-frequency HRV compared with nondiabetic controls [10].

Sympathetic overactivity and parasympathetic impairment of the autonomic system are present in premenstrual syndrome and young patients with DM [7,10]. Lightheadedness, bowel syndromes and hemodynamic instability induce dizziness or syncope related to autonomic impairment [11]. Autonomic dysfunction was noted in other psychological symptoms of depression or anxiety, and irritable bowel and bladder symptoms [12,13]. The young patients with DM had poor control because the Chinese prefer rice intake and sugary drinks (such as bubble tea) from popular beverage shops in Taiwan, and the young patients ignore the complications that may induce autonomic dysregulation. The association of diabetic females of childbearing age with PMS is worth evaluating.

The higher blood sugar causes higher progesterone through insulin stimulation during the luteal stage [5]. Autonomic dysfunction is similar in PMS and young females with DM. In the past, there was a lack of evidence about DM that is related to. We retrospectively analyzed the Taiwanese health dataset to understand young females with DM and subsequent PMS episodes.

We assumed that young female patients with DM following progesterone elevation and autonomic imbalance would have increased PMS incidence. We analyzed DM and other comorbidities for PMS to inform healthcare providers about young females with DM and related comorbid conditions to reduce PMS. Good glycemic control and parasympathetic function modification could reduce PMS by preserving autonomic function.

## 2. Materials and Methods

Taiwan National Health Insurance was started in 1995 and covered ninety-nine percent of citizens’ patients. The insurance claim data of healthcare are uploaded by medical institutions for payment. There were one million sampled patients of the National Health Insurance Research Dataset with longitudinal (LNHIRD) follow-up until 2015, and the International Classification of Disease, Tenth Revision, Clinical Modification has been fully used since 2016. The unidentified ID numbers of the dataset were set for patient privacy protection by the government. There were up to 5 inpatient disease codes in the inpatient set and up to 3 outpatient disease records in the outpatient set [14]. Our study evaluated the subsequent PMS incidence in female patients with DM using the LNHIRD from 2000 to 2015. The LNHIRD data obtained the patient’s sex, age, comorbid conditions and first diagnostic dates of DM and PMS. Our study has received approval from TSGHIRB-B-110-05.

**Figure 1:**
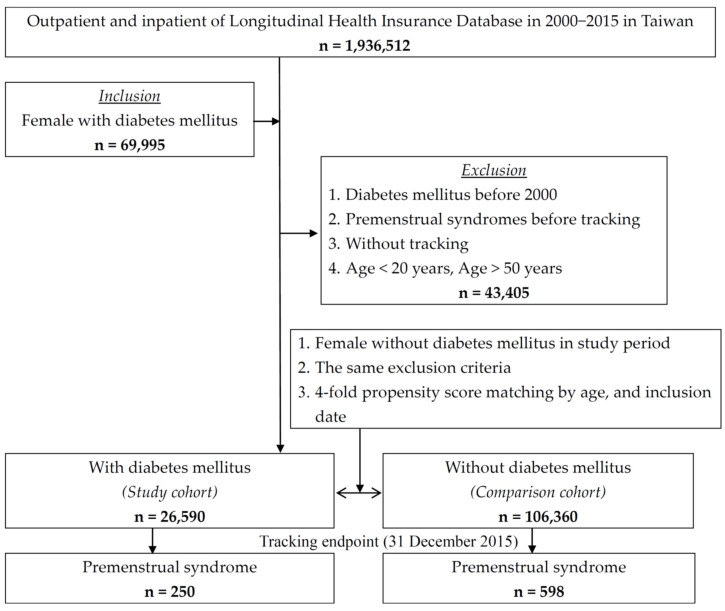
The flowchart of the study.

We retrieved newly diagnosed cases with DM by the International Classification of Disease, Ninth Revision, Clinical Modification (ICD-9-CM) disease code 250 from the LNHIRD from 2000 to 2015. The first visit date of DM was defined as the index date. We found that PMS occurrence by ICD-9-CM disease codes of 625.4 was set as the event, as in a previous study [9]. The dates of the event were the date of PMS by diagnosis or the end date of this study on 31 December 2015. The exclusion criteria were set as follows: (1) patients with DM or PMS diagnosed before 1 January 2000; (2) patients younger than 20 years old and older than 50 years old (because the majority of them were menopausal); and (3) male patients and unidentified sex patients. The control group included DM-free patients who met the exclusion criteria and were fourfold matched for age, female sex, and index date. The flowchart of this study is shown in Figure 1.

The comorbid conditions mapped by ICD-9 CM were hypertension (401–405); hyperlipidemia (272); renal disease (580–589); obesity (278),; depression (296.2–296.3, 296.82, 330.4, 331); anxiety (300.1–300.3, 300.5–300.9); palmar hyperhidrosis (780.8); irritable bowel disease (564.1); bladder disorder (596); thyrotoxicosis (242); fibromyalgia (729.1); asthma (493); chronic obstructive pulmonary disease (491, 492, 494, 496); alcohol consumption (291, 303, 305, 571.0–571.4); abortion (630–639); pregnancy (640–677); and irregular menstruation (626.4). We retrieved information on medications for DM that followed a previous study [15].

The descriptive statistics for the continuous variables were calculated by a Student’s *t* test, and the statistics for the categorical variables were calculated by the chi-squared (χ^2^) test between two groups. The cumulative incidence of PMS was drawn by the DM or DM-free group by a Kaplan–Meier curve with a log-rank test. The Cox proportional regression was analyzed for the possible risk factors for PMS with hazard ratio (HR) to show the risk. The statistical significance was set as a *p* value < 0.05. The statistical analyses were performed by SPSS software version 21 (Asia Analytics Taiwan Ltd., Taipei, Taiwan).

## 3. Results

**Figure 2:**
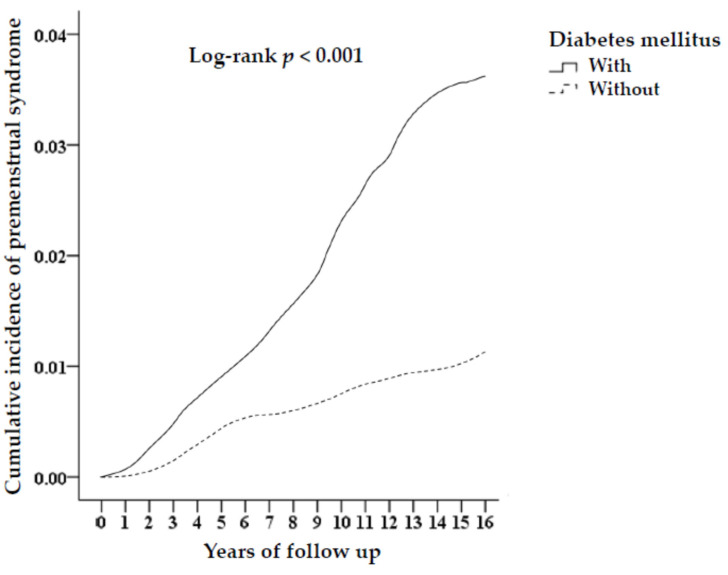
Kaplan–Meier analysis of the cumulative risk of premenstrual tension syndromes aged 20–50 years stratified by DM with the log-rank test.

There were 26,590 female patients with DM with 250 PMS (0.94%), with 104.7 every 105 person-years, and 106,360 non-DM females with 598 PMS (0.56%), with 56.91 every 105 person-years, after long-term follow-up (log-rank *p* < 0.001). The Kaplan–Meier curve stratified by DM is shown in Figure 2. The tracked time of PMS incidence was followed up with a median of 6.84 years (0.02–15.86).

The DM group had a higher PMS incidence than the DM-free group and a higher percentage of irregular menstruation, hypertension, hyperlipidemia, renal disease, depression, anxiety, irritable bowel disease, bladder disorder, greater urbanization levels, and higher hospital-level healthcare service seeking. However, patients with DM have a lower percentage of abortion and pregnancy. There were similar percentages of obesity, palmar hyperhidrosis, asthma, chronic obstructive pulmonary disease, chronic fatigue syndrome, thyrotoxicosis, fibromyalgia, and season in both groups (Table 1).

The risk for PMS in patients with DM was adjusted by a hazard ratio (HR) of 1.683 (95% confidence interval (CI): 1.104–2.124), and the other risk factors included hypertension (1.834 (95% CI: 1.246–2.498)); hyperlipidemia (1.596 (95% CI: 1.002–2.039)); renal disease (HR: 1.86 (95% CI: 1.433–2.312)); obesity (HR: 1.865 (95% CI: 1.37–2.117)); depression (HR: 2.106 (95% CI: 1.484–2.876)); anxiety (HR: 1.862 (95% CI: 1.35–2.735)); palmar hyperhidrosis (HR: 1.375 (95% CI: 1.092–1.699)); irritable bowel disease (HR: 1.444 (95% CI: 1.021–1.896)); bladder disorder (HR: 1.356 (95% CI: 1.005–1.782)); and irregular menstruation (HR: 2.301 (95% CI: 1.598–2.897)), after adjustment for other factors (Table 2).

The stratified analysis of various variables showed the occurrence of PMS in the DM group compared with the DM-free group. The risk of PMS for the patients with depression was adjusted by a HR of 1.746 (95% C.I.: 1.145–2.203, *p* < 0.001); the risk of PMS for the patients with anxiety was adjusted by a HR of 1.843 (95% C.I.: 1.209–2.326, *p* < 0.001); the adjusted HR of PMS was 2.121 (95% C.I.: 1.392–2.677, *p* < 0.001) in the patients with obesity; the adjusted HR of PMS was 1.913 (95% C.I.: 1.255–2.414, *p* < 0.001) in the patients with thyrotoxicosis; the adjusted HR of PMS was 2.21 (95% C.I.: 1.449–2.789, *p* < 0.001) in the patients with fibromyalgia; and the adjusted HR of PMS was 1.787 (95% C.I.: 1.172–2.255, *p* < 0.001) in the patients with palmar hyperhidrosis in the DM group compared with the DM-free group (Table 3).

We further stratified several DM medications and analyzed the adjusted HRs separately. The results revealed that patients treated with single-agent DM treatment with insulin (adjusted HR of 1.923 [95% C.I.: 1.261–2.423], *p* < 0.001); insulin isophane (2.935 [95% C.I.: 1.962–3.702], *p* < 0.001); insulin glargine (2.345 [95% C.I.: 1.538–2.903], *p* < 0.001); metformin (2.407 [95% C.I.: 1.438–3.038], *p* < 0.001); gliclazide (1.751 [95% C.I.: 1.123–2.209], *p* < 0.001); repaglinide (1.803 [95% C.I.: 1.185–2.077], *p* < 0.001); pioglitazone (1.733 [95% C.I.: 1.132–2.197], *p* < 0.001); nateglinide (2.251 [95% C.I.: 1.47–2.842), *p* < 0.001); mitiglinide (2.854 [95% C.I.: 1.876–3.606], *p* < 0.001); acarbose (1.452 [95% C.I.: 1.002–1.835], *p* = 0.048); exenatide (2.302 [95% C.I.: 1.533–2.952], *p* < 0.001); liraglutide (1.585 [95% C.I.: 1.036–1.996], *p* = 0.015); dulaglutide (1.552 [95% C.I.: 1.021–1.956], *p* = 0.028); sitagliptin (2.395 [95% C.I.: 1.573–3.024], *p* < 0.001); vildagliptin (2.428 [95% C.I.: 1.595–3.067], *p* < 0.001); saxagliptin (2.425 [95% C.I.: 1.592–3.025], *p* < 0.001); linagliptin (1.696 [95% C.I.: 1.113–2.141], *p* < 0.001); dapagliflozin (1.573 [95% C.I.: 1.034–1.989], *p* = 0.017); or empagliflozin (1.59 [95% C.I.: 1.042–2.007], *p* = 0.007) had higher adjusted HRs than those in the control group. Patients who were treated with a combination medical treatment with repaglinide/metformin (1.793 [95% C.I.: 1.172–2.267), *p* < 0.001); sitagliptin/metformin (2.35 [95% C.I.: 1.564–3.01], *p* < 0.001); vildagliptin/metformin (2.201 [95% C.I.: 1.573–3.034, *p* < 0.001); saxagliptin/metformin (2.156 [95% C.I.: 1.892–3.359], *p* < 0.001); or linagliptin/metformin (2.175 [95% C.I.: 1.412–2.733], *p* < 0.001) also had higher adjusted HRs than those in the control group. However, insulin lispro, insulin aspart, insulin glulisine, insulin detemir, glimepiride, miglitol, alogliptin, glimepiride/metformin and glyburide/metformin were not significantly different in terms of adjusted HRs (Table 4).

## 4. Discussion

This is the first report exploring the increased risk of PMS in females with DM. Hyperglycemia-induced insulin secretion through the central pathway increased progesterone and neuropathy development. Young female diabetic patients with possible higher progesterone and autonomic impairment carry a subsequent PMS risk [5,7]. Diabetic patients need aggressive glycemic control to decrease insulin-related hyper-progesterone and prevent nerve complications with autonomic dysfunction.

The sympathetic nerve from the T12-L1 nerve and sacral parasympathetic nerves make up the pelvic splanchnic nerve which controls the ovaries. Increased blood glucose levels trigger the activation of glucose- excited (GE) neurons in the ventromedial hypothalamus by insulin and leptin which in turn activate the sympathetic nervous system, leading to increased insulin sensitivity and glucose uptake in brown adipose tissue, the heart and skeletal muscles [16]. Type 2 DM in youth patients with menstrual dysfunction was associated with lower estradiol, free androgen index, and sex hormone-binding globulin levels [17]. Abnormal ovarian function in a type 2 DM mouse model triggered apoptosis of the granulosa cells [18]. Women with type 2 DM have reduced estrogen levels converted from androgen [19]. Insulin can modulate GnRH receptors to increase luteinizing hormone (LH) secretion, and insulin and LH enhance steroidogenesis with progesterone secretion in the corpus luteum [5,6]. Insulin not only regulates energy homeostasis but also affects the reproductive axis [5]. A study from India found a higher mean ovarian volume in women with DM than in controls [20]. The lower total antral follicle count decreased in all age groups and ovarian volume increased in women aged 20–29 years with type 2 DM compared with the healthy control group [21]. Progesterone was not significantly different between DM patients and healthy controls because the sample was not checked in the luteal stage. A previous study found lower estrogen and higher progesterone during the early luteal stage in premenstrual dysphonic syndrome (PMDD) with psychological conditions than in normal controls [22]. Higher caloric intake and progesterone in the luteal phase in patients with PMS lacked a negative relationship with leptin and ghrelin in PMS-free controls [23].

Depression and irregular menstruation were most related to PMS with a 2-fold risk adjusted for other factors. The common symptoms of PMS included these two symptoms before the doctor visited for the diagnosis. The moods of women are sensitive to fluctuations in hormone levels during the menstrual cycle. Serotonin plays an important role in mood status and fluctuations can trigger PMS symptoms. The symptoms of PMS related to serotonin deficiency enhanced the sensitivity to progesterone in women and caused irregular menstruation [2]. PMS with mental symptoms of anxiety and depression is called PMDD and has a prevalence of approximately 1.3–5.3% [1]. A meta-analysis found reduced parasympathetic activity in depression and anxiety with a higher cardiovascular risk [12]. The same finding was noted in PMS with depression [24].

PMS affects renin-aldosterone system-induced fluid overload and edema [1]. Fluid retention was combined with a hyper-sympathetic and hypo-parasympathetic nervous system in patients with chronic kidney disease [25]. This study noted that renal disease carried a risk of HR 1.86 of PMS.

Weight gain increased PMS prevalence [26]. The patients with PMS increased caloric intake during the luteal phase compared with normal controls [23]. Obese patients who experience obstructive apnea combined with an exercise intolerance having sympathetic overactivity [27]. Insulin levels rise and insulin sensitivity decreases with obesity [28]. A previous study found that obesity was associated with a 1.386 risk of PMS [9]. This study observed an increased 1.865 risk of PMS in the obesity population. The potential reason for this is DM with higher blood sugar carries a higher PMS risk. Patients with palmar hyperhidrosis carried a 1.375-fold higher risk than 1.276 in a past study [9]. The potential reason was that DM itself made some contributions to PMS.

Patients with irritable bowel disease had a 44.4% increased risk of PMS. Nearly 30% of irritable bowel disease patients suffer from constipation during the luteal phase [29]. The significant decreases in time domain and high-frequency power measured HRV in IBS patients compared with healthy controls who had lower parasympathetic tone in the meta-analysis [30]. A past study found that the mean number of bleeding days in the menstrual cycle, the severity on the PMS by scale, limb edema, depression, insomnia, and daytime sleepiness were significantly greater in the group with IBS than in the non-IBS group with PMS [31].

High blood sugar causes bladder dysfunction in DM. The autonomic and somatic nervous systems in diabetic cystopathy were affected by hyperglycemia. The slow bladder capacity increase and urinary retention were caused by bladder sensation impairment [32]. The diabetic bladder dysfunction found detrusor contractility altered with parasympathetic impairment [33]. Diabetic neuropathy with autonomic impairment causes lower urinary tract symptoms, rather than HbA1c being a predictor in females with DM [34].

Patients with PMS who live in urban areas and are concerned about their health go to higher status hospitals (medical centers and regional hospitals) for help with higher risk. Females suffer from greater work-related and life stress, stimulating the hypothalamus–pituitary gland–adrenal axis from the amygdala nucleus with sympathetic hyperactivity [35].

A past study found parasympathetic overactivity in asthma that may balance autonomic function and parasympathetic dysfunction in chronic obstructive pulmonary disease [36,37]. Potential reasons for suitable therapy include diagnosis by codes in the claim dataset that are not associated with PMS. Sympathetic overactivity in thyrotoxicosis and fibromyalgia [38,39] was noted to be related to PMS with a lower prevalence in this study.

PMS was due to the hormone imbalance during the luteal phase rather than an ambient temperature change that was associated with the season. Women with diabetes have oligomenorrhea in Korea and India [5]. There was a risk of infertility with HR 1.2 in type 2 DM compared with non-DM in the nurse study [40]. Estrogen can protect against atherogenesis and lower estrogen levels induce atheroma [19]. There were higher risks of congenital malformation, stillbirth, and perinatal death in infants of mothers with type 2 DM than in the general population [41]. Diabetic pregnancies have a higher risk in Asian women than in Caucasian women [42]. Macrosomia and giving birth to premature infants are greater risks for diabetic mothers compared with mothers without DM [43]. Diabetic females have a higher risk of delivering premature infants and those who carefully consider this are associated with a lower rate of pregnancy and abortion. The pregnant women were followed in obstetric outpatient appointments without a menstrual cycle.

Different DM medications were also utilized to stratify and analyze (Table 4). Sulfonylurea and meglitinide promoted insulin secretion [44] and carried a PMS risk. Metformin and thiazolidinedione increased insulin sensitivity and carried a higher PMS risk. Metformin also acts on the adenosine monophosphate-activated protein kinase (AMPK) pathway and inhibits the follicular secretion of androgen and aromatase to estrogen. This occurs with an LH surge with increased progesterone [45]. Metformin carries a higher risk because it influences sex hormones, similar to those in individuals with PMDD (22). Thiazolidinediones, glucagon-like peptide-1 agonists, and dipeptidyl peptidase-4 inhibitors alongside combination medical treatment are second-line treatments for DM, meaning advanced DM leads to a higher PMS risk. In addition, the α-glucosidase inhibitor sodium-glucose cotransporter 2 inhibitors decreased glucose reuptake and were associated with a milder PMS risk.

Glimepiride can induce insulin secretion. It can also increase glucose uptake in muscle and lipid tissue and inhibit glycogenosis which reduces PMS with good glycemic reduction [44]. Miglitol does not enhance the secretion of insulin but reversibly inhibits the alpha-glucosidase bound on the intestinal mucosa, slows down the time of glucose absorption into the blood, and achieves a hypoglycemic effect [44]. Insulin carries a PMS risk [5]; however, rapid-acting insulin has a short-term effect on reducing blood sugar, insulin detemir has a long half-life (21 h) and metabolites with action do not increase PMS risk.

Alogliptin and glyburide/metformin insignificantly increased PMS risk. The potential reasons are: the glycemic control of the patients was mixed with good and bad control, more detail about dose, duration of treatment, patient’s compliance and the severity of DM. Knowledge of blood sugar level in these treatment populations is needed to survey the PMS risk in the future. This result suggests that clinicians should choose glimepiride, glimepiride/metformin, rapid-acting insulin, insulin detemir, or miglitol to avoid increasing PMS risk in patients with DM. They should also understand the physiopathology of hyperglycemia-induced insulin secretion connected between energy homeostasis and the central role of reproduction.

Patients with severe PMS symptoms should consult a gynecologist or psychiatrist for treatment. PMS patients need lifestyle adjustments, such as eating healthy foods with less oil, salt and sugar; avoiding coffee; not smoking or drinking alcohol; reducing mental stress; and getting enough sleep and regular aerobic physical activity [1]. Somatic symptoms can be lessened with pain relievers. Antidepressants such as selective serotonin reuptake inhibitors can relieve psychiatric symptoms which also improves underlying serotonin deficiency-induced progesterone hypersensitivity [2]. Contraceptive pills can reduce sex hormone fluctuation [46]. GnRH inhibition can reduce ovarian function with PMS improvement, but physicians need to be aware of the risks of osteoporosis and cardiovascular disease. Edema with breast and abdominal bloating can be treated with diuretics [1]. Sympathetic activation is noted in dynamic exercise and parasympathetic activation is apparent in the recovery phase and as a result of static exercise [47]. The meta-analysis showed a decreased high frequency of HRV after endurance and supervised exercise in type 2 DM [48]. The patients with PMS received pilates exercises with the intention of relieving their symptoms [49].

This study strengthens the relationship between DM and PMS in a population follow-up study. There are some limitations in this study. First, Chinese women are often hesitant to see a doctor for PMS. This has a lower incidence than in Western countries. Past studies on PMS have been conducted in Western countries [50]. Second, this study surveyed the claim dataset rather than prospectively collected data because the autonomic function test and blood sugar were unavailable. These need to be collected in future studies to confirm our hypothesis. Third, information regarding stress, a family history of PMS, lifestyle, and nutritional factors were unavailable. These factors also affect PMS. This study used disease codes for PMS, and information on PMS severity was not available. Different PMS intensities need to be confirmed by symptom scores in DM patients. A registration study is needed. Fourth, our study analyzed patients of Chinese ethnicity in Taiwan; however, more ethnicities require studying in the future to confirm our findings. 

## 5. Conclusions

These findings indicate that PMS risk increased in young female patients with DM. This was the first exploration of young females with DM increasing PMS incidents. We reviewed past studies and emphasized parasympathetic impairment and hyperglycemia in PMS that is induced by young female patients with DM. The same sympathovagal and progesterone imbalance was noted in these two diseases. Aggressive glycemic control could reduce hyperglycemia and subsequent autonomic dysfunction. A lifestyle modification with parasympathetic revision by aerobic exercise may improve the hypo-parasympathic condition of PMS. This needs future confirmation.

## Figures and Tables

**Table 1:** Characteristics of the study at baseline.

Variables	Total (132,950)	Diabetes Mellitus (26,590)	Diabetes Mellitus-Free (106,360)	*p*
Age	29.94 ± 19.00	29.77 ± 18.45	29.98 ± 19.13	0.107
Abortion	953 (0.72%)	59 (0.22%)	894 (0.84%)	<0.001 *
Pregnancy	17,915 (13.47)	1618 (6.08%)	16,297 (15.32%)	<0.001 *
Irregular menstruation	8223 (6.19%)	1798 (6.76%)	6425 (6.04%)	<0.001 *
Hypertension	27,188 (20.45%)	5973 (22.46%)	21,215 (19.95%)	<0.001 *
Hyperlipidemia	14,102 (10.61%)	3204 (12.05%)	10,898 (10.25%)	<0.001 *
Renal disease	15,993 (12.03%)	3789 (14.25%)	12,204 (11.47%)	<0.001 *
Obesity	1252 (0.94%)	274 (1.03%)	978 (0.92%)	0.095
Depression	4471 (3.36%)	1456 (5.48%)	3015 (2.83%)	<0.001 *
Anxiety	5211 (3.92%)	1976 (7.43%)	3235 (3.04%)	<0.001 *
Palmar hyperhidrosis	15,252 (11.47%)	3132 (11.78%)	12,120 (11.4%)	0.079
Irritable bowel disease	2614 (1.97%)	579 (2.18%)	2035 (1.91%)	0.006 *
Bladder disorder	9044 (6.8%)	1979 (7.44%)	7065 (6.64%)	<0.001 *
Asthma	11,919 (8.97%)	2399 (9.02%)	9520 (8.95%)	0.715
COPD	14,368 (10.81%)	2896 (10.89%)	11,472 (10.79%)	0.625
Alcohol consumption	6444 (4.85%)	1432 (5.39%)	5012 (4.71%)	<0.001
Chronic fatigue syndrome	561 (0.42%)	120 (0.45%)	441 (0.41%)	0.398
Thyrotoxicosis	144 (0.11%)	33 (0.12%)	111 (0.1%)	0.404
Fibromyalgia	159 (0.12%)	29 (0.11%)	130 (0.12%)	0.621
Season				0.999
Spring (Mar–May)	29,310 (22.05%)	5862 (22.05%)	23,448 (22.05%)	
Summer (Jun–Aug)	32,490 (24.44%)	6498 (24.44%)	25,992 (24.44%)	
Autumn (Sep–Nov)	35,515 (26.71%)	7103 (26.71%)	28,412 (26.71%)	
Winter (Dec–Feb)	35,635 (26.8%)	7127 (26.8%)	28,508 (26.8%)	
Urbanization level				<0.001 *
1 (The highest)	35,770 (26.9%)	7961 (29.94%)	27,809 (26.15%)	
2	42,596 (32.04%)	8702 (32.73%)	33,894 (31.87%)	
3	24,626 (18.52%)	3925 (14.76%)	20,701 (19.46%)	
4 (The lowest)	29,958 (22.53%)	6002 (22.57%)	23,956 (22.52%)	
Hospital levels				<0.001 *
Medical center	41,984 (31.58%)	9689 (36.44%)	32,295 (30.36%)	
Regional hospital	47,807 (35.96%)	8933 (33.6%)	38,874 (36.55%)	
Local hospital	43,159 (32.46%)	7968 (29.97%)	35,191 (33.09%)	

* *p* < 0.05.

**Table 2:** Risk factor for premenstrual syndrome in 20- to 50-year-olds.

	Crude Hazard Ratio	*p*	Adjusted Hazard Ratio	*p*
Diabetes mellitus	1.976 (95% CI: 1.483–2.43)	<0.001 *	1.683 (95% CI: 1.104–2.124)	<0.001 *
Age	1.074 (95% CI: 0.897–1.286)	0.125	1.002 (95% CI: 0.838–1.104)	0.189
Hypertension	2.066 (95% CI: 1.472–2.705)	<0.001 *	1.834 (95% CI: 1.246–2.498)	<0.001 *
Hyperlipidemia	1.734 (95% CI: 1.086–2.271)	0.008 *	1.596 (95% CI: 1.002–2.039)	0.049 *
Renal disease	1.986 (95% CI: 1.562–2.498)	<0.001 *	1.86 (95% CI: 1.433–2.312)	<0.001 *
Obesity	2.098 (95% CI: 1.672–2.489)	<0.001 *	1.865 (95% CI: 1.37–2.117)	<0.001 *
Depression	2.482 (95% CI: 1.892–3.374)	<0.001 *	2.106 (95% CI: 1.484–2.876)	<0.001 *
Anxiety	2.335 (95% CI: 1.797–3.311)	<0.001 *	1.862 (95% CI: 1.35–2.735)	<0.001 *
Palmar hyperhidrosis	1.725 (95% CI: 1.206–2.201)	<0.001 *	1.375 (95% CI: 1.092–1.699)	0.003 *
Irritable bowel disease	1.505 (95% CI: 1.099–2.68)	0.002 *	1.444 (95% CI: 1.021–1.896)	0.037 *
Bladder disorder	1.482 (95% CI: 1.086–2.607)	0.008 *	1.356 (95% CI: 1.005–1.782)	0.046 *
Asthma	1.532 (95% CI: 0.986–2.151)	0.064 *	1.204 (95% CI: 0.725–1.808)	0.257
COPD	1.45 (95% CI: 0.824–2.033)	0.178	1.186 (95% CI: 0.689–1.762)	0.301
Alcohol consumption	1.59 (95% CI: 0.637–2.895)	0.374	1.452 (95% CI: 0.532–2.608)	0.482
Chronic fatigue syndrome	2.065 (95% CI: 0.456–4.986)	0.571	1.863 (95% CI: 0.381–4.01)	0.659
Thyrotoxicosis	2.095 (95%CI: 0.208–4.862)	0.795	1.895 (95% CI: 0.589–2.98)	0.488
Fibromyalgia	2.798 (95% CI: 0.413–5.707)	0.661	2.235 (95% CI: 0.795–4.801)	0.296
Abortion	1.489 (95% CI: 1.003–1.677)	0.047 *	1.289 (95% CI: 0.864–1.486)	0.135
Irregular menstruation	2.573 (95% CI: 1.721–3.052)	<0.001 *	2.301 (95% CI: 1.598–2.897)	<0.001 *
Season				
Spring	Reference		Reference	
Summer	1.102 (95% CI: 0.815–1.488)	0.187	1.072 (95% CI: 0.733–1.402)	0.246
Autumn	1.209 (95% CI: 0.906–1.529)	0.106	1.145 (95% CI: 0.798–1.496)	0.231
Winter	1.304 (95% CI: 0.922–1.631)	0.089	1.189 (95% CI: 0.812–1.581)	0.195
Urbanization level				
1 (The highest)	1.798 (95% CI: 1.302–2.248)	<0.001 *	1.688 (95% CI: 1.242–2.03)	<0.001 *
2	1.7 (95% CI: 1.256–2.103)	<0.001 *	1.571 (95% CI: 1.153–1.989)	<0.001 *
3	1.35 (95% CI: 0.917–1.725)	0.094	1.263 (95% CI: 0.842–1.677)	0.188
4 (The lowest)	Reference		Reference	
Hospital levels				
Medical center	2.384 (95% CI: 1.562–2.971)	<0.001 *	1.781 (95% CI: 1.267–2.392)	<0.001 *
Regional hospital	1.897 (95% CI: 1.35–2.602)	<0.001 *	1.702 (95% CI: 1.245–2.379)	<0.001 *
Local hospital	Reference		Reference	

* *p* < 0.05.

**Table 3:** The stratified analysis of various variables showed the occurrence of PMS in the DM group compared with the DM-free group.

Diabetes Mellitus	With			Without (Reference)			With vs. Without (Reference)			
Stratified	Events	PYs	Rate (per 10^5^ PYs)	Events	PYs	Rate (per 10^5^ PYs)	Adjusted Hazard Ratio	95% CI		*p*
Total	250	238,778.20	104.70	598	1,050,836.80	56.91	1.683	1.104–2.124		<0.001 *
Abortion										
Without	217	238,088.94	91.14	445	1,041,811.56	42.71	1.952	1.280–2.463		<0.001 *
With	33	689.26	4787.74	153	9025.24	1695.25	2.583	1.695–3.260		<0.001 *
Pregnancy										
Without	250	222,076.09	112.57	598	889,782.91	67.21	1.683	1.104–2.124		<0.001 *
With	0	16,702.11	0.00	0	161,053.89	0.00	-	-	-	-
Irregular menstruation										
Without	229	222,281.98	103.02	559	986,301.58	56.68	1.663	1.091–2.098		0.008 *
With	21	16,496.22	127.30	39	64,535.22	60.43	1.927	1.264–2.432		<0.001 *
Obesity										
Without	198	236,346.09	83.78	507	1,040,966.56	48.70	1.573	1.032–1.986		0.039 *
With	52	2432.11	2138.06	91	9870.24	921.96	2.121	1.392–2.677		<0.001 *
Depression										
Without	222	223,797.85	99.20	560	1,012,039.32	55.33	1.640	1.076–2.070		0.012 *
With	28	14,980.35	186.91	38	38,797.48	97.94	1.746	1.145–2.203		<0.001*
Anxiety										
Without	213	220,510.29	96.59	558	1,011,046.14	55.19	1.601	1.050	2.021	0.025 *
With	37	18,267.91	202.54	40	39,790.66	100.53	1.843	1.209–2.326		<0.001 *
Thyrotoxicosis										
Without	249	238,422.09	104.44	596	1,049,347.24	56.80	1.682	1.103–2.123		<0.001 *
With	1	356.11	280.81	2	1489.56	134.27	1.913	1.255–2.414		<0.001 *
Fibromyalgia										
Without	249	238,509.48	104.40	596	1,049,538.58	56.79	1.682	1.103–2.122		<0.001 *
With	1	268.72	372.13	2	1298.22	154.06	2.21	1.449–2.789		<0.001 *
Hyperlipidemia										
Without	214	209,714.96	102.04	531	942,957.55	56.31	1.658	1.087–2.092		0.008 *
With	36	29,063.24	123.87	67	107,879.25	62.11	1.824	1.197–2.302		<0.001 *
Hypertension										
Without	177	184,806.09	95.78	478	840,858.54	56.85	1.541	1.011–1.945		0.041 *
With	73	53,972.11	135.26	120	209,978.26	57.15	2.165	1.420–2.732		<0.001 *
Renal disease										
Without	212	204,636.54	103.60	527	929,302.92	56.71	1.671	1.096–2.109		0.002 *
With	38	34,141.66	111.30	71	121,533.88	58.42	1.743	1.143–2.199		<0.001 *
Asthma										
Without	221	217,119.09	101.79	544	956,705.55	56.86	1.637	1.074–2.067		0.013 *
With	29	21,659.11	133.89	54	94,131.25	57.37	2.135	1.401–2.694		<0.001 *
Chronic obstructive pulmonary disease										
Without	219	212,727.22	102.95	532	937,434.51	56.75	1.659	1.089–2.094		0.007 *
With	31	26,050.98	119.00	66	113,402.29	58.20	1.870	1.227–2.360		<0.001 *
Alcohol consumption										
Without	236	225,892.81	104.47	569	1,001,258.53	56.83	1.682	1.103–2.122		<0.001 *
With	14	12,885.39	108.65	29	49,578.27	58.49	1.699	1.115–2.144		<0.001 *
Palmar hyperhidrosis										
Without	218	210,293.64	103.66	529	930,863.97	56.83	1.669	1.095–2.106		0.003 *
With	32	28,484.56	112.34	69	119,972.83	57.51	1.787	1.172–2.255		<0.001 *
Chronic fatigue syndrome										
Without	248	237,610.80	104.37	595	1,046,400.56	56.86	1.679	1.101–2.119		0.001 *
With	2	1167.40	171.32	3	4436.24	67.62	2.317	1.520–2.925		<0.001 *
Irritable bowel disease										
Without	244	233,542.76	104.48	586	1,030,661.84	56.86	1.681	1.103–2.121		<0.001 *
With	6	5235.44	114.60	12	20,174.96	59.48	1.763	1.156–2.224		<0.001 *
Bladder disorder										
Without	230	222,650.40	103.30	555	980,858.55	56.58	1.670	1.095–2.108		0.002 *
With	20	16,127.80	124.01	43	69,978.25	61.45	1.846	1.211–2.330		<0.001 *
Season										
Spring	52	53,538.72	97.13	154	276,378.06	55.72	1.594	1.046–2.012		0.025 *
Summer	60	58,486.21	102.59	140	246,702.65	56.75	1.654	1.085–2.087		0.008 *
Autumn	66	63,095.11	104.60	153	266,866.38	57.33	1.669	1.095–2.106		0.002 *
Winter	72	63,658.16	113.10	151	260,889.71	57.88	1.788	1.173–2.256		<0.001 *
Urbanization level										
1 (The highest)	77	71,148.62	108.22	158	274,940.60	57.47	1.723	1.130–2.174		<0.001 *
2	83	77,964.33	106.46	184	323,037.12	56.96	1.710	1.122–2.158		<0.001 *
3	37	35,318.49	104.70	118	208,137.00	56.69	1.689	1.108–2.132		<0.001 *
4 (The lowest)	53	54,346.76	97.56	138	244,722.08	56.39	1.583	1.038–1.997		0.039 *
Level of hospital										
Medical center	95	87,013.02	109.18	183	319,153.14	57.34	1.742	1.143–2.198		<0.001 *
Regional hospital	83	80,029.67	103.71	219	382,464.29	57.26	1.657	1.087–2.091		0.007 *
Local hospital	72	71,735.51	100.37	196	349,219.37	56.13	1.636	1.073–2.065		0.012 *

* *p* < 0.05.

**Table 4:** The stratified analysis of various medications for diabetes mellitus showed the occurrence of PMS and risk of PMS compared with the DM-free group.

DM Subgroups	Populations	Events	PYs	Rate (per 10^5^ PYs)	Adjusted HR (95% C.I.)	*p*
Without DM	106,360	598	1,050,836.8	56.91	Reference	
With DM	26,590	250	238,778.2	104.7	1.683 (1.104–2.124)	<0.001 *
Without medication	8722	81	78,315.22	103.43	1.663 (1.091–2.098)	<0.001 *
With medication	17,868	169	160,462.98	105.32	1.694 (1.111–2.138)	<0.001 *
Insulin	2794	30	25,083.21	119.6	1.923 (1.261–2.423)	<0.001 *
Rapid-acting insulin						
Insulin + lispro	30	0	259.44	0	0	0.984
Insulin + aspart	534	4	4789.21	83.52	1.343 (0.881–1.682)	0.189
Insulin + glulisine	8	0	48.25	0	0	0.977
Intermediate-acting insulin						
Insulin + isophane	61	1	537.36	186.09	2.935 (1.962–3.702)	<0.001 *
Long-acting insulin						
Insulin + glargine	228	3	2056.22	145.90	2.345 (1.538–2.903)	<0.001 *
Insulin + detemir	1013	6	9182.11	65.34	1.05 (0.689–1.322)	0.378
Biguanides						
Metformin	501	3	2003.4	149.75	2.407 (1.438–3.038)	<0.001 *
Sulfonylureas						
Gliclazide	1431	14	12,855.14	108.91	1.751 (1.123–2.209)	<0.001 *
Glimepiride	1689	14	15,160.21	92.35	1.484 (0.972–1.873)	0.084
Thiazolidinedione						
Repaglinide	798	9	8009.72	112.36	1.803 (1.185–2.077)	<0.001 *
Pioglitazone	310	3	2778.03	107.99	1.733 (1.132–2.197)	<0.001 *
Meglitinides						
Nateglinide	167	2	1428.23	140.03	2.251 (1.47–2.842)	<0.001 *
Mitiglinide	133	2	1125.06	177.77	2.854 (1.876–3.606)	<0.001 *
α-glucosidase inhibitor						
Acarbose	739	6	6633.22	90.45	1.452 (1.002–1.835)	0.048 *
Miglitol	137	0	1235.1	0	0	0.986
Glucagon-like peptide-1 agonist						
Exenatide	307	4	2746.89	145.62	2.302 (1.533–2.952)	<0.001 *
Liraglutide	229	2	2035.14	98.27	1.585 (1.036–1.996)	0.015 *
Dulaglutide	116	1	1032.29	96.87	1.552 (1.021–1.956)	0.028 *
Dipeptidyl peptidase-4 inhibitor						
Sitagliptin	103	2	1342.18	149.01	2.395 (1.573–3.024)	<0.001 *
Vildagliptin	221	3	1986.24	151.04	2.428 (1.595–3.067)	<0.001 *
Saxagliptin	205	3	1988.30	150.88	2.425 (1.592–3.025)	<0.001 *
Alogliptin	120	1	1080.33	92.56	1.488 (0.998–1.879)	0.051
Linagliptin	231	2	1895.22	105.53	1.696 (1.113–2.141)	<0.001 *
Sodium-Glucose Cotransporter 2 Inhibitors						
Dapagliflozin	569	5	5098.43	98.07	1.573 (1.034–1.989)	0.017 *
Empagliflozin	227	2	2021.22	98.95	1.59 (1.042–2.007)	0.007 *
Combination medical treatment						
Glimepiride + Metformin	1525	13	15,160.33	85.75	1.378 (0.903–1.735)	0.102
Glyburide + Metformin	1797	15	16,132.1	92.98	1.495 (0.986–1.886)	0.064
Repaglinide + Metformin	889	9	8066.57	111.57	1.793 (1.172–2.267)	<0.001 *
Sitagliptin + Metformin	150	2	1348.25	148.34	2.35 (1.564–3.01)	<0.001 *
Vildagliptin + Metformin	148	2	1340.22	149.23	2.201 (1.573–3.034)	<0.001 *
Saxagliptin + Metformin	125	2	1025.11	195.1	2.156 (1.892–3.359)	<0.001 *
Linagliptin + Metformin	333	4	2980.25	134.22	2.175 (1.412–2.733)	<0.001 *

* *p* < 0.05; PYs: person-years; HR: hazard ratio; C.I.: confidence interval.

## Data Availability

Restrictions apply to the availability of these data. Data were obtained from the National Health Insurance database and are available from the authors with the permission of the National Health Insurance Administration of Taiwan.
